# gp3tools: An R Package for Reproducible Analysis and Reporting of Gazepoint GP3 Eye-Tracking Exports

**DOI:** 10.3390/jemr19040076

**Published:** 2026-07-14

**Authors:** Stefanos Balaskas

**Affiliations:** eGovernment & eCommerce Lab (Innovation & Entrepreneurship), Department of Business Administration, University of Patras, 26504 Patras, Greece; s.balaskas@ac.upatras.gr

**Keywords:** eye tracking, eye movements, Gazepoint GP3, R package, reproducible research, pupil preprocessing, area of interest, fixation analysis, research software, workflow evaluation

## Abstract

Reproducible eye-movement research requires a documented path from device exports to structured, quality-checked, and reportable data objects. Gazepoint GP3 and Gazepoint Analysis provide accessible gaze, fixation, pupil, timing, media, and area-of-interest data, but their folder-based CSV exports are not automatically analysis-ready. Existing R tools support important stages of eye-tracking and pupillometry analysis, but they generally assume that data have already been organised into suitable structures; they do not provide a Gazepoint-aware workflow beginning with export-folder checks, all-gaze/fixation pairing, sampling and tracking-quality diagnostics, and preservation of preprocessing decisions through reporting. This article presents gp3tools, an open-source R package (R version 4.6.1) that converts Gazepoint GP3/Gazepoint Analysis exports into structured R objects, diagnostic summaries, preprocessing outputs, model-ready tables, interoperability objects, and reproducible reports. Rather than introducing a new statistical estimator, the package provides an export-aware workflow scaffold for import checking, quality control, pupil preprocessing, area-of-interest, fixation and transition summaries, model preparation, interoperability, and reporting. A synthetic Gazepoint-style demonstration dataset was used to evaluate workflow execution without exposing private participant data. The demonstration identified all expected file pairs and produced sample-level gaze/pupil tables, fixation tables, sampling-quality summaries, area-of-interest summaries, review flags, and reporting outputs. A small, private real-export compatibility check further showed that the workflow could process one empirical Gazepoint folder without manual restructuring. These results support a bounded software-evaluation claim: gp3tools executes the tested Gazepoint-style workflow and returns expected diagnostic and reporting objects, but the evidence does not establish hardware accuracy, preprocessing accuracy, computational scalability, general robustness across all Gazepoint exports, or substantive psychological or perceptual effects.

## 1. Introduction

Eye-movement research increasingly depends on computational workflows that transform device-specific exports into interpretable measures of gaze allocation, fixation behaviour, pupil dynamics, and area-of-interest (AOI) engagement [1,2]. These outcomes are not extracted from an eye tracker as neutral analytical facts. They are constructed through technical and methodological decisions involving calibration quality, sampling behaviour, validity flags, fixation parsing, pupil preprocessing, AOI definitions, temporal windows, aggregation rules, exclusion criteria, and statistical model specification. Reproducibility therefore depends not only on the reported model but also on the visibility and repeatability of the preprocessing and quality-control pathway that precedes it [3,4].

This issue is especially important in applied eye-tracking settings, where researchers often work with vendor-exported files rather than standardised research-data objects. Gazepoint GP3 and Gazepoint Analysis provide accessible exports containing gaze coordinates, fixation summaries, pupil measurements, timing fields, media identifiers, and AOI labels [3,4]. These exports are useful for behavioural, marketing, human–computer interaction, usability, and communication research, but they are not automatically analysis-ready. A typical project must still verify file completeness and pairing, inspect sampling and validity, assess AOI labels and denominators, document preprocessing choices, and generate reproducible analysis tables [5,6].

The need for explicit preprocessing is well established. Pupil-size analyses are sensitive to blinks, artefacts, interpolation, smoothing, and baseline correction, and preprocessing choices can affect downstream inference [7,8,9]. AOI analyses similarly depend on spatial definitions, sample- or fixation-assignment rules, and denominator construction for proportion- or dwell-based outcomes [10]. Fixation, pupil, and AOI outcomes therefore require reporting practices that make preprocessing assumptions inspectable rather than implicit.

Existing R tools address important parts of this workflow. eyetrackingR supports preparation, summarisation, visualisation, and analysis of eye-tracking data in R [11]. PupillometryR provides a dedicated pipeline for preparing, plotting, and analysing pupillometry data [12]. General R packages support reproducible documents, statistical graphics, linear and generalised linear mixed models, generalised additive models, and model reporting [13,14,15,16]. These tools are valuable, but they are mostly platform-agnostic or focused on later analytical stages. Gazepoint users still need an export-aware workflow that begins with the concrete structure of GP3/Gazepoint Analysis folders and carries the data through import checks, preprocessing, diagnostics, model preparation, and reporting within a single R environment.

For this reason, gp3tools was implemented as a separate package rather than as an extension of an existing framework. Extending eyetrackingR or PupillometryR would not directly address Gazepoint-specific file discovery, all-gaze/fixation pairing, summary-file handling, export-structure diagnostics, or reporting of vendor-specific preprocessing decisions. Similarly, pymovements provides a broader Python framework for eye-movement processing, whereas gp3tools targets R users who need a Gazepoint-specific import, quality-control, interoperability, and reporting layer before downstream modelling or visualisation. The contribution is therefore not a replacement of existing analytical packages, but a device/export-aware workflow layer for making Gazepoint-specific preprocessing decisions explicit before later analysis begins.

gp3tools was developed to address this methodological and practical gap. The package converts Gazepoint-style export folders into structured R objects, diagnostic summaries, model-ready tables, visual outputs, reporting checklists, and reproducible reports. Its purpose is not to introduce a new statistical estimator, propose a universal theory of eye-movement preprocessing, or replace established modelling packages. Instead, it provides infrastructure for documenting and inspecting the steps through which Gazepoint exports become analysis inputs.

This article presents the design, scope, software-evaluation strategy, and demonstration workflow of gp3tools. The package provides an import and file-checking layer for Gazepoint all-gaze, fixation, and summary exports; workflow components for sampling diagnostics, tracking quality, pupil preprocessing, AOI summaries, transition structures, model-ready data preparation, exclusion recommendations, and reporting readiness; and public reproducibility material, including synthetic Gazepoint-style exports, vignettes, tests, R Markdown workflows, and a Zenodo-archived software release. The demonstration evaluates software behaviour and workflow execution under controlled conditions; it is not used to draw substantive psychological, perceptual, or marketing conclusions.

## 2. Methodological Background and Related Software

Eye-tracking is widely used across psychology, neuroscience, human–computer interaction, information systems, marketing, usability research, and applied behavioural science [17,18,19]. Its value lies in recording temporally precise indicators of overt visual attention and converting gaze behaviour into measures such as fixations, dwell time, scanpaths, pupil responses, and area-of-interest engagement [20]. These measures, however, depend on calibration quality, sampling behaviour, validity filtering, fixation detection, spatial assignment, temporal aggregation, and statistical modelling. As Duchowski [18] notes, eye tracking can support diagnostic and interactive applications, but inference depends on the quality and interpretation of the recorded signal.

This issue is especially relevant for applied studies using accessible or low-cost devices. Gazepoint GP3 has been evaluated as a low-cost eye tracker suitable for information-systems research when stimuli are designed with the device’s constraints in mind [21]. For the present article, the central problem is narrower than general eye-tracking methodology: researchers working with Gazepoint exports need a reproducible route from vendor-generated CSV folders to structured, quality-checked, analysis-ready R objects without relying on manual file inspection, spreadsheet editing, or one-off preprocessing scripts.

Several methodological issues recur across eye-movement workflows. Data quality should be reported explicitly, with users able to inspect quality metrics across preprocessing stages [22]. AOI-based analysis involves spatial classification uncertainty, because AOI size and placement can affect false-negative and false-positive assignments [23]. Pupil-size analysis also requires careful preprocessing because pupil responses are sensitive to luminance, near fixation, arousal, task demands, artefacts, interpolation, smoothing, and baseline correction [7,8,9,24]. Together, these issues support software workflows that preserve quality-control decisions, AOI denominators, artefact flags, interpolation decisions, and derived outcomes as inspectable objects.

Existing software addresses important parts of this landscape. In R, eyetrackingR supports preparation, summarisation, visualisation, and analysis of eye-tracking data, whereas PupillometryR provides a dedicated pipeline for pupil-size data. In Python, pymovements provides a broader framework for parsing, event detection, event-property extraction, visualisation, public datasets, documentation, and testing [25]. Kassner et al. [26] similarly illustrate the value of open eye-tracking ecosystems through the Pupil platform. These tools are important, but their primary contribution is not Gazepoint-specific export-folder evaluation. Gazepoint users must still identify all-gaze, fixation, and summary files; check participant-level pairing; harmonise gaze, pupil, fixation, media, timing, validity, and AOI fields; inspect sampling and tracking quality; preserve preprocessing decisions; and produce reportable audit outputs. These tasks can be handled with custom scripts, but they remain fragmented without a dedicated Gazepoint-aware workflow layer.

Table 1 positions gp3tools relative to related tools. The aim is not to rank software quality, but to clarify the package’s methodological niche. Existing tools provide valuable general eye-tracking, pupillometry, modelling, or open-platform functionality. gp3tools contributes the export-aware layer needed to connect Gazepoint GP3/Gazepoint Analysis CSV folders to structured R objects, quality-control outputs, preprocessing branches, model-ready datasets, interoperability structures, and reporting material.

The contribution of gp3tools is therefore deliberately specific. It does not compete with general-purpose eye-tracking or pupillometry packages as a universal analysis environment. Instead, it provides a Gazepoint-specific workflow layer for researchers who work with GP3/Gazepoint Analysis CSV exports and need a reproducible route from export folders to validated master tables, pupil summaries, AOI outputs, fixation summaries, transition structures, model-ready datasets, diagnostic plots, reporting checklists, and reproducible reports. Without such a layer, the same workflow would require researchers to combine manual folder inspection, custom import scripts, separate quality-control code, independent pupil and AOI preprocessing routines, ad hoc exclusion documentation, and separate reporting scripts. gp3tools makes these steps explicit, testable, and repeatable within one R-based workflow. This design is consistent with research-software principles. Wilson et al. argue that scientific software should be treated as part of the research apparatus and should be version-controlled, tested, documented, and automated. Smith et al. establish the importance of persistent software citation, while Katz et al. [27] extend FAIR principles to research software. gp3tools applies these principles to the practical problem of Gazepoint-based eye-movement analysis [28].

## 3. Materials and Methods

### 3.1. Package Scope and Design Objectives

gp3tools is an open-source R package for working with Gazepoint GP3 and Gazepoint Analysis CSV exports. Its scope is intentionally bounded: the package targets desktop-style Gazepoint export folders containing all-gaze, fixation, summary, media, pupil, timing, and area-of-interest (AOI) information. It is not designed as a universal eye-tracking platform, an independent hardware-validation tool, or a replacement for established statistical modelling packages.

The package was developed to provide a documented route from vendor-style exports to structured analysis objects. Five design principles guided the implementation: import checks should precede analysis; preprocessing decisions should remain visible; outputs should retain structured status information; model-ready datasets should be produced without imposing a single inferential framework; and reporting should be supported through saved tables, plots, checklists, vignettes, and R Markdown outputs.

gp3tools is distributed under the MIT licence. The manuscript refers to version 1.0.1 unless otherwise stated. The development repository is available at https://github.com/stefanosbalaskas/gp3tools, and the versioned release is archived on Zenodo under DOI 10.5281/zenodo.20731340. The repository includes source code, function documentation, tests, vignettes, example datasets, citation metadata, and synthetic Gazepoint-style demonstration resources, consistent with principles of inspectable, testable, citable, and reproducible scientific software [19,27,29].

The intended users are applied eye-movement researchers who work with Gazepoint exports and need a transparent preprocessing and reporting workflow before statistical analysis. These include behavioural scientists, consumer and marketing researchers, human–computer interaction researchers, usability laboratories, teaching laboratories, and methodologically oriented users.

### 3.2. Gazepoint Export Structure and Supported Data Types

Gazepoint GP3 and Gazepoint Analysis projects commonly generate multiple CSV files rather than a single tidy research dataset. These files may include participant-level all-gaze exports, fixation exports, and summary outputs. All-gaze files typically contain timestamps, media identifiers, gaze coordinates, best-point-of-gaze variables, pupil-size variables, validity indicators, and AOI labels. Fixation files provide event-level information such as fixation location, onset, duration, and media identifiers. Summary files may contain higher-level recording or media summaries. The exact export structure can vary across projects, software settings, and analysis configurations.

gp3tools treats this folder-based structure as part of the analytic problem. The package identifies expected export types, checks file-pair completeness, imports all-gaze and fixation data, and harmonises available participant, media, trial, time, gaze, pupil, validity, fixation, and AOI fields into structured R objects. These objects support sampling diagnostics, tracking-quality checks, pupil preprocessing, AOI summaries, fixation summaries, transition analyses, and model preparation.

The package does not assume that a valid-looking CSV file is suitable for inference. Import is followed by checks for expected columns, duplicate keys, missing fields, file-pair inconsistencies, sampling irregularities, tracking-quality problems, and review-required recordings. This approach is consistent with the view that low-cost systems such as Gazepoint GP3 can support useful research workflows when their constraints are acknowledged [21]. gp3tools addresses the complementary software problem: how to make the exported data inspectable before analysis. The public repository includes synthetic Gazepoint-style demonstration exports that make the workflow executable and reviewable without redistributing private participant recordings.

### 3.3. Software Architecture and Workflow Families

gp3tools is organised around workflow families rather than isolated utility functions. Each family corresponds to a recurrent problem in moving from Gazepoint GP3/Gazepoint Analysis exports to analysis objects: file discovery and pairing, master-table validation, sampling and tracking-quality diagnostics, pupil preprocessing, AOI/fixation/transition workflows, model preparation, interoperability, and reporting. Table 2 summarises these families as a functional map rather than a complete function index; detailed function-level documentation is provided in the package help files and repository.

The methodological rationale is that preprocessing and diagnostic choices should be explicit and study-specific. File-pair checks identify missing or unmatched exports before analysis. Sampling and tracking-quality summaries expose possible recording problems without imposing universal exclusion rules. Pupil workflows preserve distinctions among raw, cleaned, interpolated, baseline-corrected, and window-level values, following recommendations that pupil preprocessing should document artefacts, missingness, interpolation, and baseline correction [8,9]. AOI, fixation, and transition workflows preserve spatial labels, denominators, event summaries, and transition structures because AOI assignment depends on spatial definitions and classification uncertainty [10]. Model-preparation functions produce inputs for established R modelling tools, including mixed-effects, count, and smooth time-course workflows [15,16,30], without prescribing a single statistical model.

Table 3 summarises the rationale, inspected outputs, and limitations of selected diagnostic decisions. The thresholds and preprocessing branches are implemented as configurable decision aids, not universal standards.

**Figure 1:**
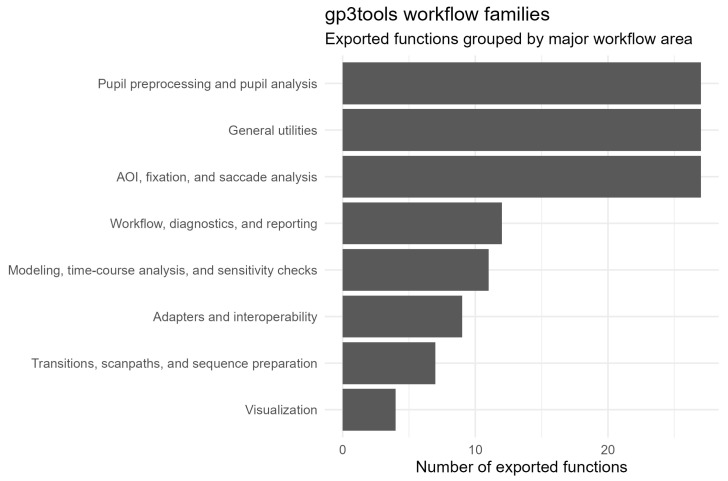
gp3tools workflow families. Exported functions grouped by major workflow area. The figure summarises the package architecture and should be interpreted as a functional map rather than a performance result.

Figure 1 provides a compact overview of the main workflow families implemented in gp3tools. It should be interpreted as a map of workflow coverage, not as evidence of methodological superiority or empirical performance.

### 3.4. Synthetic Demonstration Dataset

A synthetic Gazepoint-style demonstration dataset was used to evaluate package behaviour under reproducible and shareable conditions. The dataset was designed to mimic realistic Gazepoint export folders while avoiding release of private participant recordings. It includes synthetic all-gaze files, fixation files, a summary export, media identifiers, AOI states, gaze coordinates, pupil values, validity indicators, missing pupil intervals, invalid samples, and participant-level quality variation.

The demonstration dataset serves a software-evaluation purpose rather than an empirical research purpose. It allows users and reviewers to inspect whether the package can discover expected files, validate file pairs, estimate sampling behaviour, construct master tables, flag review-required recordings, summarise AOI outcomes, process pupil data, prepare model-ready objects, generate diagnostic outputs, and create reports. The synthetic values are not interpreted as psychological, perceptual, marketing, usability, or clinical findings.

Synthetic data make the demonstration executable, redistributable, and reviewable, and allow controlled insertion of missing pupil intervals, invalid samples, AOI states, fixation files, and quality variation. However, they cannot fully reproduce calibration failures, participant movement, incomplete sessions, unexpected export settings, stimulus-specific visual structure, lighting effects, or laboratory-specific file naming. Therefore, the synthetic demonstration evaluates workflow execution and diagnostic object generation, not general robustness across all real Gazepoint projects.

To complement the public synthetic demonstration, gp3tools was also checked on a small private Gazepoint export folder containing real device exports. This compatibility check included six all-gaze files, six fixation files, and four summary files, producing 7340 all-gaze rows, 337 fixation rows, and a diagnostic report. Because these recordings were private and were not collected for public software benchmarking, they are not redistributed and are reported only in aggregate. This check provides limited evidence that the workflow can execute on one real Gazepoint export structure, but it does not replace broader empirical validation across laboratories, tasks, participants, devices, or export settings. This compatibility check was not designed to represent the diversity of empirical Gazepoint use cases. It does not cover multiple laboratories, experimental paradigms, stimulus types, AOI densities, recording durations, calibration conditions, participant behaviours, export settings, or Gazepoint software versions. Its role is therefore restricted to demonstrating that the workflow can execute on one real export structure in addition to the public synthetic demonstration. Broader empirical validation would require a larger set of real export folders, ideally contributed by different laboratories and representing different recording conditions and study designs.

### 3.5. Software Evaluation and Quality-Assurance Strategy

The software evaluation strategy combines automated tests, reproducible examples, synthetic demonstration data, a private real-export compatibility check, package checks, documentation, continuous integration, and release archiving. The term evaluation is used deliberately. The manuscript does not claim to validate preprocessing accuracy, fixation-classification accuracy, pupil-recovery accuracy, AOI-assignment validity, hardware accuracy, or statistical-model correctness. Instead, the evaluation assesses whether predefined workflow components execute as intended, return structured outputs, preserve diagnostic information, and expose review-required cases.

Quality assurance was implemented at three levels. At the package level, functions were tested using automated unit tests and standard R package checks. At the workflow level, synthetic Gazepoint-style exports exercised import, file-structure validation, preprocessing, model-preparation, plotting, reporting, and export branches. At the compatibility level, the private real-export folder tested whether the same high-level workflow could process real Gazepoint all-gaze, fixation, and summary files without manual restructuring. At the dissemination level, the package was documented through function help, README guidance, vignettes, example datasets, citation metadata, and a versioned Zenodo release. This strategy follows principles that scientific software should be checked, documented, preserved, and citable [19,27,29].

Table 4 distinguishes the level of evidence available for the main workflow components. Several components are software-tested and workflow-demonstrated but not independently validated against external ground-truth eye-tracking data.

Automated tests cover core workflow branches, including import behaviour, file-pair validation, master-table diagnostics, sampling summaries, tracking-quality outputs, pupil preprocessing, AOI workflows, transition workflows, model-preparation helpers, optional-dependency handling, reporting utilities, and structured error/status reporting. The workflow evaluation focused on whether expected files were detected and paired; whether sample-, fixation-, pupil-, AOI-, and transition-level objects were returned with structured status information; whether diagnostic and reporting outputs were generated without manual intervention; and whether limitations or review-required cases were preserved rather than silently removed.

Available quantitative software-quality indicators were limited to package-testing and package-check results. The package test suite completed with 6788 passing expectations, and R CMD check completed with 0 errors, 0 warnings, and 0 notes. These metrics support package integrity, example consistency, and standard R-package compliance, but they are not measures of preprocessing accuracy, code coverage, runtime scalability, memory performance, or comparative speed against other packages. Formal benchmarking of code coverage, execution time, memory use, and performance on very large Gazepoint projects remains a target for future development.

The evaluation claims are deliberately narrow. The synthetic demonstration shows that gp3tools can execute an end-to-end Gazepoint-like workflow and return expected diagnostic, analysis, and reporting objects. The private real-export check shows that the same workflow can execute on one small empirical Gazepoint export structure. Neither source establishes general robustness across laboratories, export settings, experimental designs, or empirical data-quality profiles. Users remain responsible for inspecting calibration quality, stimulus design, missingness, validity rates, AOI definitions, preprocessing decisions, exclusion rules, and model assumptions in each empirical study.

### 3.6. Usage Example and Reproducible Demonstration Workflow

The manuscript demonstration was implemented as an executable R workflow. The workflow starts from a public synthetic Gazepoint-style export folder distributed with the package, runs the import and checking pipeline, saves diagnostic outputs, and returns a structured workflow object. This code is included to show the reproducible structure of the demonstration, not to define a required analysis protocol for all studies. It also functions as a compact usage example: users can replace the demonstration directory with their own Gazepoint export folder and then inspect the returned file-pairing, sampling, tracking-quality, pupil, AOI, fixation, transition, and reporting objects before making study-specific analysis decisions (Table 5).

For empirical projects, users should replace demo_dir with the local folder containing their own Gazepoint GP3/Gazepoint Analysis exports. Successful workflow execution should not be treated as evidence that the empirical data are automatically suitable for inference. Calibration quality, device setup, stimulus layout, validity rates, AOI definitions, preprocessing choices, exclusion criteria, and statistical assumptions must still be reported and justified for each study.

## 4. Results

The Section 4 reports software-evaluation outputs rather than empirical eye-tracking findings. The synthetic dataset was designed to exercise predefined workflow branches, including file pairing, sampling diagnostics, pupil preprocessing, AOI summaries, fixation and transition outputs, model-preparation objects, interoperability outputs, and report generation. Successful execution on these data therefore indicates that the implemented workflows return expected objects under controlled conditions; it does not independently validate preprocessing accuracy, algorithmic performance, hardware accuracy, or empirical robustness. The private real-export compatibility check in Section 4.2 provides limited evidence that the workflow can also execute on one small empirical Gazepoint export structure.

### 4.1. Import, File-Pairing, and Master-Table Validation

The synthetic showcase contained 48 all-gaze files, 48 fixation files, and one summary export. gp3tools detected all 48 expected all-gaze/fixation pairs as complete and identified no problematic file pairs. The high-level workflow produced 115,200 all-gaze rows, 5691 fixation rows, four exported CSV tables, two diagnostic plots, and an HTML diagnostic report (Table 6).

The import workflow converted the export folder into harmonised sample-level and fixation-level objects. Master-table checks confirmed that expected participant, media, timing, gaze, pupil, validity, fixation, and AOI fields were available for downstream processing. This stage treats file discovery, pairing, and structural checks as prerequisites to analysis rather than as administrative steps.

### 4.2. Private Real-Export Compatibility Check

To complement the public synthetic demonstration, the workflow was checked on a small private Gazepoint export folder containing real device exports. The folder contained six all-gaze files, six fixation files, and four summary files. gp3tools detected the expected participant-level all-gaze/fixation structure, imported 7340 all-gaze rows and 337 fixation rows, and created a diagnostic report without manual restructuring.

The real-export folder differed from the synthetic showcase in scale and structure. It contained fewer recordings, fewer summary files than participant-level all-gaze/fixation files, and a more limited empirical export structure. This provides useful compatibility evidence because the folder was not generated specifically for the manuscript demonstration.

This result should be interpreted narrowly. The private real-export check demonstrates compatibility with one small empirical Gazepoint export structure, but it does not establish robustness across all Gazepoint software versions, laboratory naming conventions, calibration conditions, recording-quality profiles, or stimulus designs. Because the recordings were private and were not collected for public software benchmarking, they are reported only in aggregate and are not redistributed. The check therefore strengthens practical credibility, but it remains compatibility evidence rather than independent validation of preprocessing accuracy or general real-world performance. Accordingly, this result should not be described as evidence of robust real-world performance. It is a compatibility check showing that the package can process one empirical export folder without manual restructuring. The result strengthens practical plausibility, but it remains narrower than validation across heterogeneous empirical datasets.

### 4.3. Sampling-Rate and Tracking-Quality Diagnostics

The workflow generated 192 sampling-rate rows and 192 tracking-quality rows, corresponding to participant-by-media diagnostic summaries. Sampling diagnostics showed a nominal 60 Hz sample stream across all four synthetic conditions. This was expected because the synthetic generator created regularly spaced samples. The sampling-rate result is therefore best interpreted as a positive workflow check rather than as independent evidence about device sampling variability.

**Figure 2:**
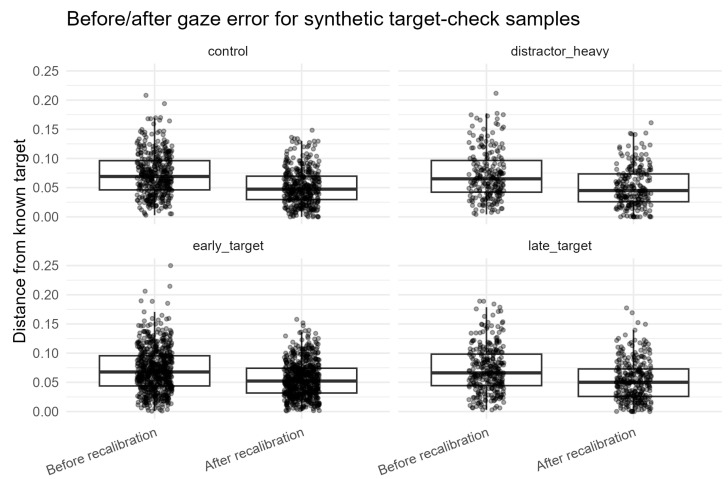
Gaze recalibration before and after correction. Boxplots and jittered points show the distance from known synthetic target-check samples before and after recalibration. The *x*-axis identifies the preprocessing stage, the *y*-axis reports target-check distance, and colours/legend entries identify the synthetic condition where applicable. The figure is based on synthetic Gazepoint-style data and demonstrates the recalibration workflow; it should not be interpreted as hardware-validation evidence.

The workflow also demonstrated gaze-signal quality checks and optional recalibration. The before/after recalibration plot shows that synthetic target-check error was lower after applying the correction step across the four synthetic conditions (Figure 2). This output demonstrates that gp3tools can represent recalibration as an auditable preprocessing operation. It does not independently validate hardware accuracy or replace empirical calibration-quality inspection.

**Figure 3:**
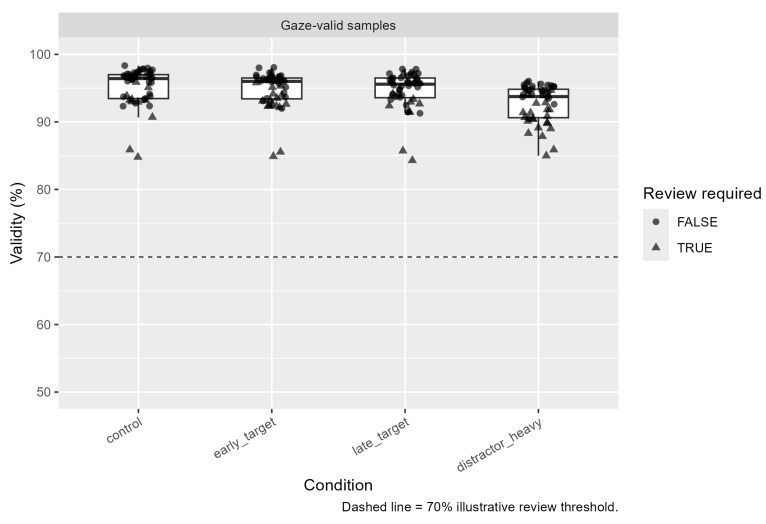
Recording-level review flags by synthetic condition.

Tracking-quality diagnostics identified 60 recording-level rows requiring review. Review flags varied across synthetic conditions, with the highest review-required rate in the distractor-heavy condition (Figure 3; Table 7). These outputs show that gp3tools preserves recording-level diagnostic information for inspection rather than reducing data quality to a single pass/fail decision.

This audit-oriented design is consistent with the data-quality reporting logic proposed by Jakobi et al. Rather than reducing eye-tracking quality to a single pass/fail decision, gp3tools returns structured diagnostic outputs that can be inspected, reported, and linked to later exclusion decisions.

### 4.4. Pupil Preprocessing and Window-Level Workflows

**Figure 4:**
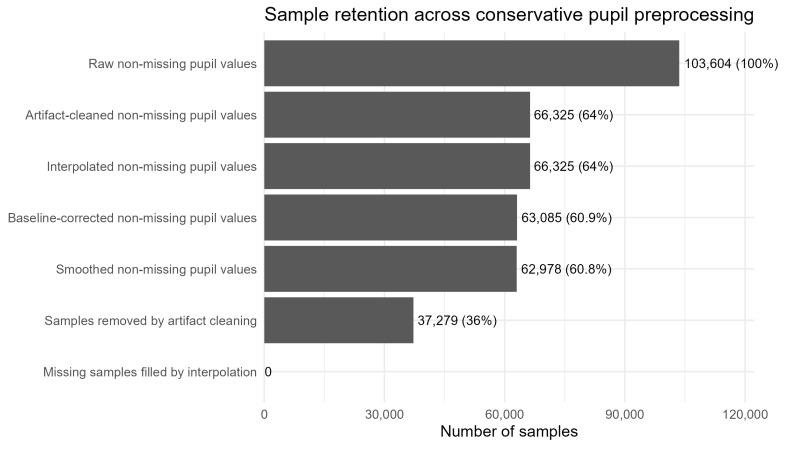
Sample retention across conservative pupil preprocessing. The conservative artefact-cleaning branch retained 66,325 of 103,604 non-missing raw pupil samples and removed 37,279 samples. Interpolation did not fill additional samples, indicating that remaining gaps were not eligible under the configured interpolation rule. Baseline correction and smoothing resulted in small additional reductions in non-missing pupil values.

The pupil workflow completed the main preprocessing branches, including artefact flagging, Hampel filtering, interpolation, PCHIP interpolation, smoothing, baseline correction, baseline auditing, gap auditing, pupil-window summaries, and model-ready pupil-window preparation (Appendix A, Figure A2). A conservative preprocessing branch retained 66,325 of 103,604 non-missing raw pupil samples and removed 37,279 samples during artefact cleaning. Interpolation did not fill additional samples, indicating that the remaining gaps were not eligible under the configured rule; baseline correction and smoothing produced small additional reductions in non-missing values (Figure 4).

**Figure 5:**
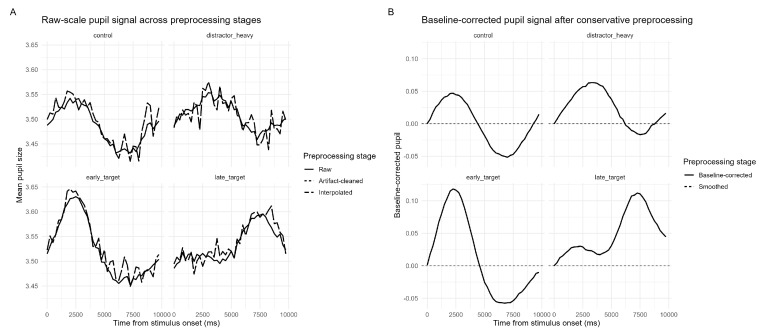
Conservative pupil preprocessing: raw-scale and baseline-corrected signals. Panel (**A**) shows raw-scale pupil trajectories before and after conservative artefact cleaning and interpolation. Panel (**B**) shows baseline-corrected and smoothed pupil trajectories. Values are averaged within 250 ms bins by synthetic condition. Axes and legends identify time, pupil scale, preprocessing branch, and synthetic condition. The figure is a synthetic workflow demonstration and does not represent an empirical pupil-response finding.

**Figure 6:**
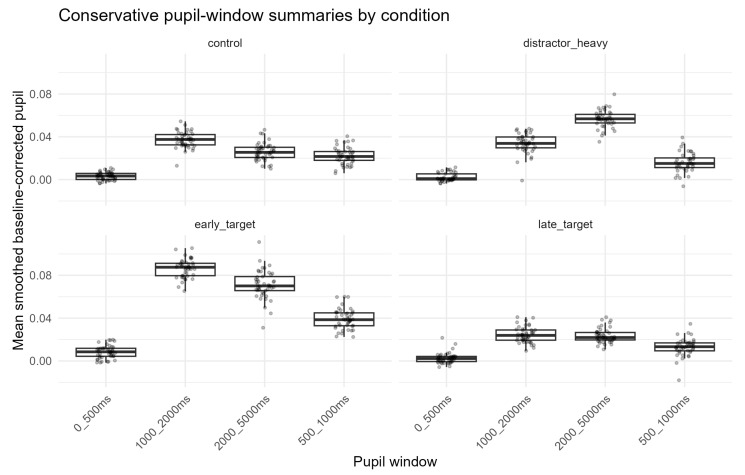
Conservative pupil preprocessing status by condition. The **left** panel shows the percentage of raw pupil samples missing before conservative preprocessing. The **right** panel shows the percentage of samples classified as unusable after conservative artefact flagging. Values are summarised by synthetic condition.

The conservative branch separated raw missingness from stricter artefact-screening decisions. Raw pupil missingness was relatively low, whereas the proportion of samples classified as unusable after conservative artefact flagging was higher (Figure 5 and Figure 6). Raw, artefact-cleaned, and interpolated trajectories remained on the original pupil-size scale, whereas baseline-corrected and smoothed trajectories were expressed as change from baseline.

**Figure 7:**
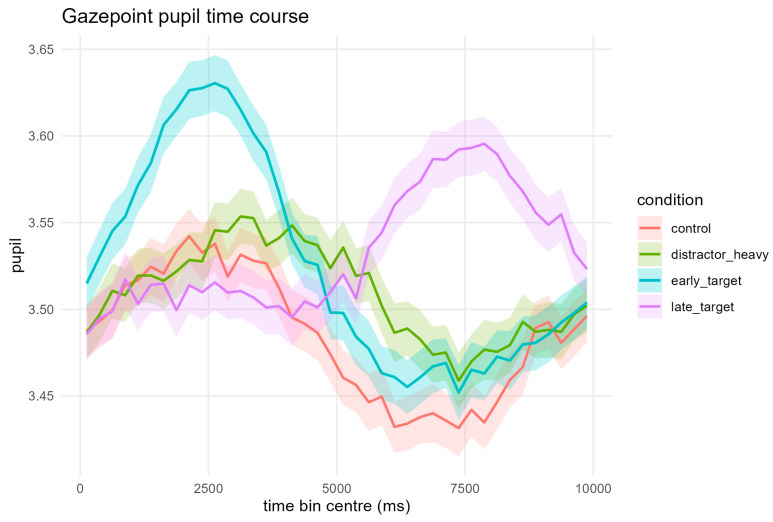
Pupil time course by synthetic condition. The figure is based on synthetic Gazepoint-style data and demonstrates time-course plotting and model-preparation outputs; it should not be interpreted as evidence of empirical pupil-response differences.

Pupil-window summaries were generated for raw, linear-interpolated, PCHIP-interpolated, and conservative branches. Each main raw/interpolated branch returned 768 window-level rows, and confirmatory model-ready pupil-window data were prepared (Table 8). Representative LMM, sensitivity, GAMM, and PFE-GAMM objects were also created (Figure 7). In this synthetic run, differences between raw, linear-interpolated, and PCHIP window means were small or negligible; this demonstrates execution of the sensitivity branch, not a general claim about interpolation effects in empirical pupil data.

These outputs show that pupil preprocessing can be represented as a structured sequence of operations. They do not establish physiological conclusions about pupil response.

### 4.5. AOI, Fixation, and Transition Workflows

**Figure A1:**
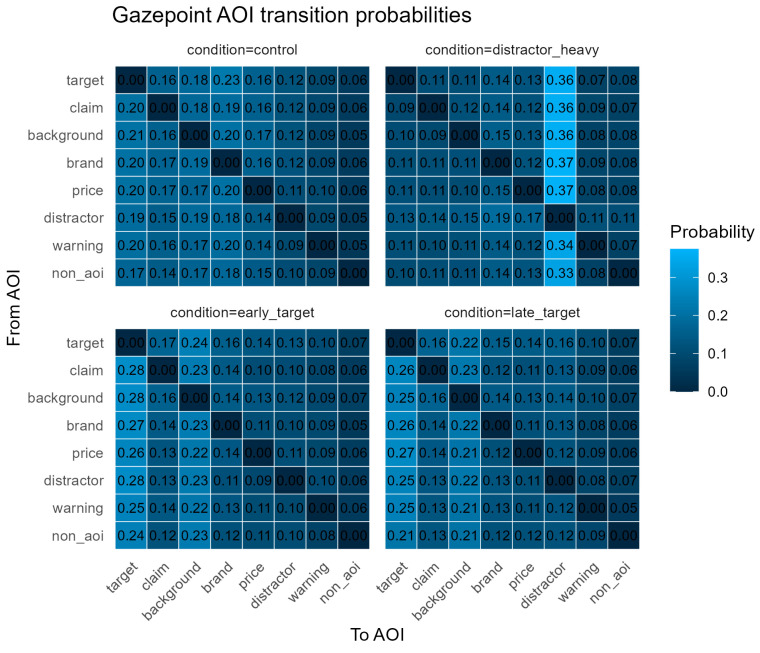
AOI transition probabilities by synthetic condition. The figure is based on synthetic Gazepoint-style data and demonstrates transition-matrix output; it should not be interpreted as an empirical scanpath result.

**Figure A2:**
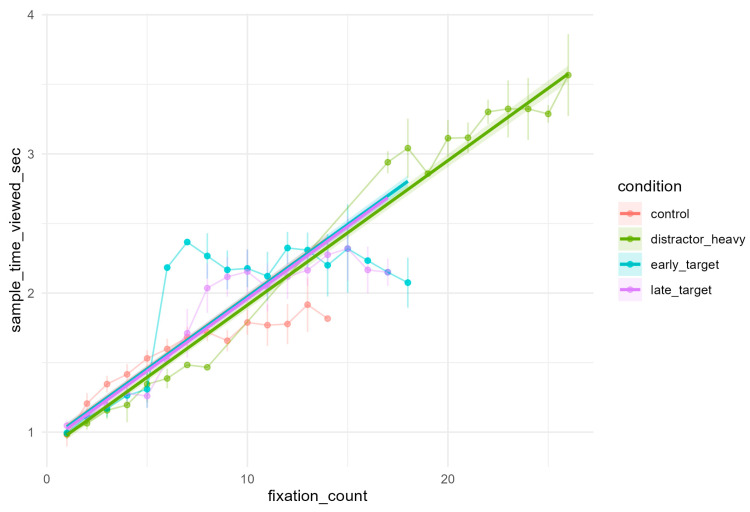
Model-implied AOI viewed-time predictions. The figure is based on synthetic Gazepoint-style data and demonstrates prediction-plot output from model-ready AOI summaries; it should not be interpreted as an empirical effect.

The AOI workflow generated 1344 AOI summary rows covering viewed time, time to first fixation, fixation count, and fixation duration. The synthetic conditions were designed to exercise AOI-window summaries, denominator auditing, AOI GLMM preparation, empirical-logit transformation, AOI time-course modelling, and transition-matrix construction. They should not be interpreted as substantive perceptual or behavioural effects (Appendix A, Figure A1).

**Figure 8:**
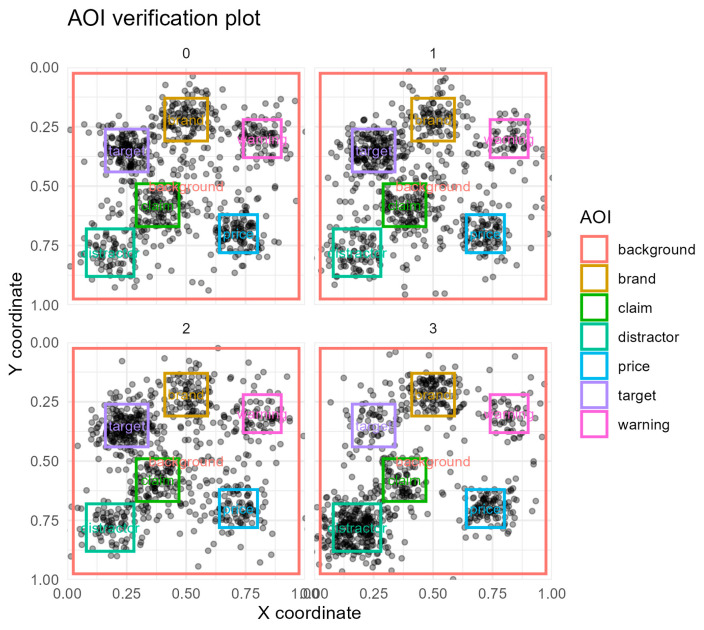
AOI geometry verification plot. The figure is based on synthetic Gazepoint-style data and is used to verify that AOI definitions and gaze samples are represented coherently; it is not an empirical AOI-validity result.

**Figure 9:**
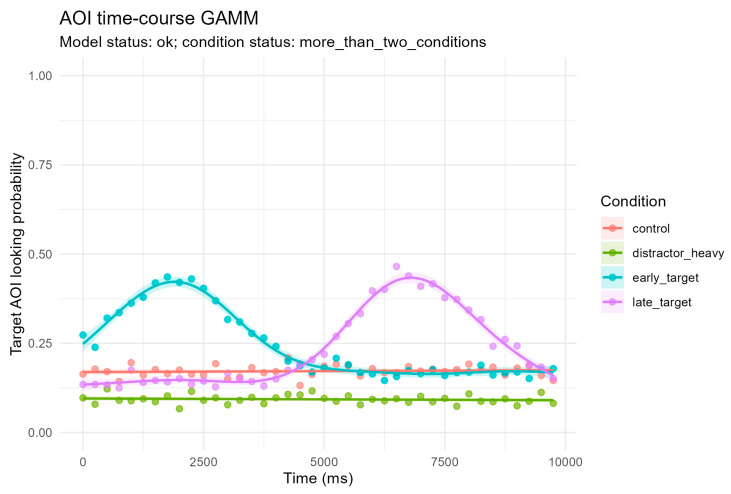
AOI target-looking trajectory by synthetic condition. The *x*-axis reports time, the *y*-axis reports model-implied target-looking probability, and the legend identifies synthetic condition. The fitted curves show the intended early-target and late-target synthetic timing patterns, whereas the control and distractor-heavy conditions show lower and flatter target-looking probabilities. The figure is based on synthetic Gazepoint-style data and is presented as a model-preparation demonstration, not as evidence of empirical attentional effects.

AOI geometry was visualised to check that gaze samples and AOI definitions were represented coherently (Figure 8). The target AOI time-course GAMM illustrates model-ready AOI time-course output, with fitted curves reflecting the intended early-target and late-target synthetic timing patterns (Figure 9). These plots are software demonstrations, not evidence of empirical attentional effects.

The fixation workflow used 5691 fixation rows across 192 participant-by-media recordings, averaging approximately 29.6 fixations per recording. The resulting structure supported fixation-trial summaries, fixation-aligned objects, AOI entries, AOI transitions, transition matrices, time-varying transition matrices, Markov-style objects, HMM-ready data, semi-Markov-ready data, and negative-binomial transition-count sensitivity (Table 9). These outputs demonstrate that AOI, fixation, and transition information can be preserved in structured forms suitable for inspection before inferential modelling.

### 4.6. Model-Ready Outputs, Reporting Readiness, and Interoperability

The demonstration also produced model-ready objects for pupil-window, AOI-window, fixation, transition, and time-course analyses. It generated model diagnostics, nested-model comparisons, prediction plots, leave-one-unit sensitivity results, divergence-point objects, preprocessing multiverse objects, and cluster-preparation data. These outputs are intended to support established R modelling tools rather than impose a single inferential framework.

The reporting workflow completed readiness gates, exclusion recommendations, reporting checklists, design-balance audits, condition-quality imbalance audits, exclusion-flow audits, post-exclusion balance checks, and an analysis-decision audit. The interoperability workflow generated adapter outputs for eyetools, gazer, PupillometryR-style, and eyetrackingR-style data structures, with optional external-package checks for gazeR-style and eyetools fixation-detection workflows (Table 10).

### 4.7. End-to-End Synthetic Demonstration Output

The full synthetic demonstration exercised the export-to-report workflow. The showcase contained 48 synthetic all-gaze files, 48 synthetic fixation files, and one summary export. The workflow detected all expected file pairs and produced 115,200 all-gaze rows, 5691 fixation rows, 192 sampling-quality rows, 1344 AOI summary rows, and 60 review-required rows (Table 11). These are software-demonstration results, not empirical evidence about attention, perception, pupil response, usability, or consumer behaviour.

The software-evaluation evidence should be interpreted narrowly. Following Wilson et al. [19], Smith et al. [29], and Katz et al. [27], the package uses testing, documentation, versioning, citation metadata, and archived releases to support reproducible scientific software. These materials show that gp3tools can execute its intended Gazepoint-style workflow and return expected diagnostic, analysis, and reporting objects under tested conditions. They do not establish preprocessing accuracy, algorithmic performance, hardware accuracy, or general robustness across empirical Gazepoint recordings. Users remain responsible for inspecting calibration quality, stimulus design, missingness, validity rates, AOI definitions, preprocessing decisions, exclusion rules, and model assumptions for each study. Figure 2, Figure 3, Figure 4, Figure 5, Figure 6, Figure 7, Figure 8 and Figure 9 should be interpreted in the same bounded manner. They illustrate that the plotting, preprocessing, AOI, fixation, transition, recalibration, and model-preparation branches produce interpretable outputs from the synthetic demonstration data. They do not evaluate algorithmic accuracy against external ground truth and should not be read as evidence of empirical gaze, pupil, AOI, or scanpath effects.

### 4.8. Software-Quality Evidence

The demonstration was accompanied by package-level and workflow-level software-quality evidence. Automated tests evaluated core functions, edge cases, optional-dependency paths, and structured workflow outputs. Package checks evaluated installation, documentation, examples, vignettes, and R CMD check compliance. Cross-platform continuous integration assessed behaviour across major operating systems and R release/development settings. Release archiving provided a persistent software citation through Zenodo. Documentation, example data, and synthetic exports were used to make the workflow inspectable by users and reviewers (Table 12).

## 5. Discussion

### 5.1. Contribution to Reproducible Eye-Movement Research

This article presented gp3tools as a Gazepoint-specific R workflow layer for converting GP3/Gazepoint Analysis exports into structured objects, diagnostics, preprocessing outputs, and reporting material. Its contribution is infrastructural rather than theoretical or statistical. The package is intended to make preprocessing and quality-control decisions more visible than they may be in local spreadsheets, ad hoc scripts, or undocumented manual checks. This should be understood as a design objective and workflow affordance, not as empirical evidence that gp3tools improves reproducibility relative to existing workflows.

The results show that gp3tools can preserve intermediate objects, status columns, diagnostic summaries, and reporting outputs within one R workflow. This is most relevant for applied laboratories, teaching settings, usability groups, and small interdisciplinary teams that work with Gazepoint exports but do not maintain dedicated data-engineering pipelines. In such contexts, the challenge is not only statistical modelling but also documenting how raw exports become analysis inputs. Sampling-rate summaries, gaze-validity summaries, review flags, pupil-window diagnostics, AOI-denominator checks, and exclusion recommendations support inspectable quality-assurance practice. They do not, however, demonstrate improved data quality, transparency, or reproducibility in a comparative empirical sense.

### 5.2. Relationship to Existing R Eye-Tracking Tools

gp3tools is best understood as a Gazepoint-specific complement to existing eye-tracking and statistical software. Packages such as eyetrackingR and PupillometryR provide tools for preparing, summarising, visualising, and analysing eye-tracking or pupil data once those data have been placed into an appropriate structure. General R packages such as lme4 [31], glmmTMB [30], and mgcv [32] provide established modelling infrastructure for mixed models, count models, and smooth time-course analyses. The role of gp3tools is to connect Gazepoint export folders to these later analytical stages.

This positioning matters because Gazepoint Analysis exports are not automatically tidy model-ready datasets. Users must still determine whether all-gaze and fixation files are paired correctly, whether expected variables are present, whether sampling and validity are plausible, whether AOI labels are usable, and whether preprocessing choices can be reconstructed later. gp3tools addresses this export-aware part of the workflow.

The package also supports interoperability rather than software isolation. Adapter outputs for eyetrackingR-style, PupillometryR-style, gazer-style, and eyetools-style workflows allow users to combine Gazepoint-specific import, validation, and diagnostic review with broader eye-tracking ecosystems. Alternative software may still be preferable when projects do not use Gazepoint exports, when data are already harmonised into a device-independent format, when the main aim is custom event detection from raw gaze streams, or when the laboratory works primarily in Python. The methodological gap claimed here is therefore based on the functional comparison in Table 1 and on the absence of Gazepoint-specific export-folder validation, all-gaze/fixation pairing, structured diagnostic reporting, and export-to-report scaffolding in the compared tools. It is not based on a benchmark showing superior speed, accuracy, or reproducibility.

### 5.3. Practical Use Cases

The main use case for gp3tools is quality-controlled import of Gazepoint GP3/Gazepoint Analysis exports. Researchers can inspect an export folder, check all-gaze/fixation pairing, create a master table, and summarise sampling and tracking quality before making analysis decisions. This is useful when multiple participant-level files must be processed consistently and when exclusion, preprocessing, and reporting decisions need to be reconstructed later.

A second use case is transparent pupil preprocessing. The package preserves distinctions among raw pupil values, artefact-cleaned values, interpolated values, baseline-corrected values, smoothed values, and window-level summaries. The conservative preprocessing branch illustrates how raw missingness, artefact handling, interpolation eligibility, and baseline correction can be reported as a sequence of inspectable operations. This aligns with recommendations that pupil analyses should document artefact handling, interpolation, smoothing, and baseline correction [8,24,33].

A third use case is AOI-centred analysis. gp3tools supports AOI geometry checks, AOI-window summaries, denominator audits, empirical-logit transformations, AOI time-course preparation, and transition matrices. This matters because AOI outcomes depend on spatial definitions, assignment rules, and denominator choices. As Orquin et al. [10] argue, AOI assignment involves classification uncertainty; therefore, AOI definitions and AOI-derived denominators should be inspectable before modelling.

A fourth use case is reporting readiness. Exclusion recommendations, reporting checklists, readiness gates, analysis-decision audits, and exportable tables help users document what was inspected, flagged, transformed, and generated. This is relevant for manuscripts, Supplementary Materials, preregistration updates, teaching examples, internal laboratory reports, and reproducibility reviews.

### 5.4. Interpretation of the Synthetic Demonstration

The synthetic demonstration should be interpreted as a controlled workflow test. It shows that gp3tools can execute the intended Gazepoint-style pipeline and return expected objects, diagnostics, plots, summaries, and reports. It does not provide evidence about psychological attention, perception, consumer behaviour, pupil physiology, usability, clinical outcomes, hardware accuracy, or the quality of empirical Gazepoint recordings.

The value of the synthetic dataset is that it is public, redistributable, and able to exercise controlled missingness, invalid samples, AOI states, pupil variability, fixation files, and quality differences. Its limitation is that it cannot reproduce the full heterogeneity of real recordings, including calibration failures, participant movement, lighting variation, incomplete sessions, stimulus-specific visual structure, or laboratory-specific export conventions. Researchers using gp3tools with empirical data must therefore continue to justify calibration procedures, device setup, AOI definitions, preprocessing rules, exclusion criteria, and statistical assumptions.

### 5.5. Limitations

Several limitations should be noted. First, gp3tools is intentionally Gazepoint-specific. This focus is useful for GP3/Gazepoint Analysis CSV exports, but it limits direct applicability to other eye-tracking systems without export conversion or adapter workflows. Future changes in Gazepoint export conventions, column names, timing fields, AOI labels, or summary-file structures may require package updates and renewed compatibility testing.

Second, the package does not independently validate hardware accuracy. Sampling-rate diagnostics, gaze-validity summaries, recalibration checks, and target-error plots can help users inspect exported data, but they do not replace formal device-validation studies. Users working with small AOIs, dense visual layouts, mobile stimuli, or high-precision spatial hypotheses must still consider device accuracy, calibration quality, participant positioning, screen geometry, and stimulus design. This is particularly important for low-cost devices, as discussed by [21].

A third priority is broader empirical testing across real Gazepoint export folders. Such testing should include multiple laboratories, experimental paradigms, stimulus designs, AOI layouts, recording durations, calibration-quality profiles, and Gazepoint software versions. These tests need not always require redistribution of participant-level data, but they should document export variations, uncommon column structures, naming differences, missing-file patterns, timing-field differences, and edge cases that require package updates. Public benchmark workflows and redistributable empirical case-study datasets would further strengthen independent replication where ethical and privacy constraints permit sharing. Formal benchmarking of runtime, memory use, code coverage, and scalability is also needed, particularly for large Gazepoint projects and high-density AOI designs.

Fourth, gp3tools does not implement a new built-in fixation-detection algorithm. The fixation workflow is designed primarily for vendor-exported fixation files, Gazepoint Analysis events, or externally prepared fixation/event structures. Users who require custom fixation parsing, velocity-based event detection, or device-independent event classification should use specialised fixation-detection tools and treat gp3tools as an import, validation, summarisation, interoperability, or reporting layer where appropriate.

Fifth, performance with very large datasets and high-density AOI designs has not yet been systematically benchmarked. The package was tested through automated tests, synthetic demonstrations, and a small private real-export compatibility check, but large multi-session projects may place greater demands on memory, plotting, transition-matrix construction, AOI-denominator auditing, and model-preparation workflows.

Sixth, generalizability to other low-cost eye trackers is limited. The methodological principles underlying gp3tools—file validation, quality-control reporting, preprocessing traceability, and reproducible reporting—may be relevant beyond Gazepoint, but the implemented import logic, column harmonisation, naming assumptions, and workflow checks were designed for Gazepoint GP3/Gazepoint Analysis exports. Other low-cost trackers would require device-specific adapters, export-conversion layers, and separate validation.

Seventh, the demonstration relies primarily on synthetic data, complemented by one small private real-export compatibility check. This makes the workflow public, reproducible, and inspectable, but it limits the empirical scope of the results. Synthetic workflow evaluation cannot reproduce the full heterogeneity of real Gazepoint studies, including calibration failures, participant movement, blink structure, lighting variation, incomplete sessions, dense AOI layouts, long recordings, laboratory-specific naming conventions, or changing export settings. The private real-export check provides limited evidence that the workflow can execute on one empirical export structure, but it does not establish general performance across laboratories, tasks, participants, devices, software versions, or experimental paradigms.

Eighth, some advanced workflows depend on optional external packages or on modelling assumptions from other R packages. Users should still inspect model convergence, singularity, overdispersion, smooth specification, random-effect structure, and sensitivity results when conducting inferential analyses. Interoperability also requires maintenance because adapter outputs must remain aligned with changes in external package expectations, data structures, and modelling conventions.

### 5.6. Future Development

Future development should prioritise documentation, interoperability, empirical workflow examples, and benchmarking rather than uncontrolled expansion of analytical scope. Practical vignettes, case-study templates, manuscript-reporting examples, and decision guides would help users understand when to use sampling diagnostics, pupil preprocessing, AOI summaries, transition matrices, GAMM preparation, or reporting checklists.

A second priority is continued interoperability. Because eye-tracking laboratories often combine multiple packages, gp3tools should continue to provide adapter outputs for related R eye-tracking tools while preserving Gazepoint-specific validation. Additional examples could show complete workflows in which gp3tools performs Gazepoint import, quality control, and reporting, while specialised packages perform downstream pupil, AOI, time-course, or model-specific analyses.

A third priority is broader empirical testing across real Gazepoint export folders. Such testing need not require redistribution of participant data, but it can identify export variations, uncommon column structures, naming differences, and edge cases that require package updates. Compatibility testing should also be repeated when Gazepoint software releases change export structure, column naming, AOI parsing, timing fields, or summary-table formats. Formal benchmarking of runtime, memory use, code coverage, and scalability is also needed, particularly for large Gazepoint projects and high-density AOI designs. A public real-data vignette would further strengthen independent replication if a redistributable Gazepoint dataset becomes available.

Finally, gp3tools should remain a workflow scaffold for Gazepoint data rather than becoming an unbounded collection of statistical methods. Maintaining this boundary will make the package easier to test, document, cite, and use in reproducible eye-movement research.

## 6. Conclusions

This article introduced gp3tools, an R package for working with Gazepoint GP3 and Gazepoint Analysis exports in R. The package addresses a practical gap in applied eye-movement research: the need to move from vendor-specific CSV export folders to structured, quality-checked, model-ready, and reportable objects through a documented workflow.

The evidence reported in this manuscript supports a bounded software-evaluation claim. Automated tests, package checks, the synthetic demonstration, and one small private real-export compatibility check show that gp3tools can execute the intended Gazepoint-style workflow and return expected diagnostic, preprocessing, modelling, interoperability, and reporting outputs under tested conditions. These results do not establish preprocessing accuracy, hardware accuracy, algorithmic superiority, computational scalability, or robust performance across heterogeneous empirical Gazepoint projects.

The contribution of gp3tools is methodological and infrastructural. It does not replace study-specific calibration checks, empirical data-quality assessment, AOI-definition decisions, preprocessing justification, or statistical model evaluation. Instead, it provides a structured R-based framework for making these decisions easier to inspect, document, and communicate. Software workflows can support transparent practice, but they cannot remove the need for domain expertise, device-specific judgement, and study-specific methodological justification.

Future development should focus on broader real-export compatibility testing, empirical case-study templates, clearer decision guides, integration examples with established eye-tracking and modelling tools, and formal benchmarking of runtime, memory use, code coverage, and scalability. Possible extensions to other eye-tracking systems should be approached cautiously through adapters or export-conversion layers rather than by diluting the package’s Gazepoint-specific design.

Within these boundaries, gp3tools provides a practical and citable R-based workflow layer for Gazepoint eye-tracking data. Its broader contribution is to treat preprocessing, exclusion, interoperability, and reporting decisions as visible parts of the research record rather than as hidden steps between data export and inference.

## Figures and Tables

**Table 1:** Positioning of gp3tools relative to related eye-tracking software.

Feature	EyetrackingR	PupillometryR	Pymovements	Pupil Platform	gp3tools
Primary environment	R	R	Python	Hardware/software platform	R
Main orientation	General eye-tracking analysis	Pupillometry analysis	General eye-movement processing	Open-source eye-tracking ecosystem	Gazepoint GP3/Gazepoint Analysis workflow scaffold
Gazepoint export-folder discovery	Not the primary focus	Not the primary focus	Not the primary focus	Not applicable as a Gazepoint CSV workflow	Supported
All-gaze/fixation file-pair validation	Not provided as a Gazepoint-specific workflow	Not provided	Not provided as a Gazepoint-specific workflow	Not applicable	Supported
Sampling and tracking-quality diagnostics for Gazepoint exports	Requires user-defined preparation	Limited to pupil-oriented workflows after data preparation	Requires dataset-specific preparation	Platform-specific rather than Gazepoint-export specific	Supported through structured diagnostic tables and review flags
Pupil preprocessing	Limited/general	Core functionality	Supported through general processing workflows	Platform-dependent	Supported with Gazepoint-aware flags, interpolation branches, baseline auditing, and window summaries
AOI, fixation, and transition workflows	AOI/time-window analysis after preparation	Not the primary focus	General event-processing functionality	Platform-dependent	Supported for Gazepoint sample, fixation, AOI, and transition outputs
Model-ready R outputs	Supported for prepared eye-tracking data	Supported for prepared pupil data	Outside R; requires transfer to R for R-based modelling	Not the primary focus	Supported for pupil, AOI, fixation, transition, and time-course workflows
Reporting checklists and reproducible report generation	Not the primary focus	Not the primary focus	Not the primary focus	Not the primary focus	Supported
Interoperability with related R workflows	Native package	Native package	External Python ecosystem	External platform	Provides adapter-style outputs for broader R eye-tracking workflows
Unique contribution	General R analysis tools for eye-tracking data	Dedicated R pipeline for pupil-size analysis	Extensible Python framework for eye-movement processing	Open hardware/software platform for eye tracking	Gazepoint-specific export-to-report workflow for validation, quality control, preprocessing, model preparation, and reporting

**Table 2:** Main workflow families supported by gp3tools.

Workflow Family	Purpose	Example Outputs
Import and file inspection	Read Gazepoint-style exports and identify expected file types.	Classified files; imported all-gaze, fixation, and summary tables
Master-table validation	Create and inspect analysis-ready combined data.	Validated master tables; column diagnostics; duplicate-key checks
Sampling and tracking quality	Check sampling behaviour, gaze validity, and review flags.	Sampling-rate summaries; tracking-quality tables; review flags
Pupil preprocessing	Prepare pupil data for window and time-course analysis.	Baseline-corrected pupil series; interpolation flags; pupil-window summaries
AOI workflows	Summarise and audit gaze behaviour by area of interest.	AOI sample summaries; fixation summaries; transition matrices; denominator audits
Model preparation and sensitivity	Prepare model-ready datasets and evaluate robustness.	LMM, GLMM, and GAMM input tables; leave-one-out summaries; divergence-point results
Reporting and export	Generate reporting checklists, exports, and reproducibility outputs.	Reporting checklists; exclusion recommendations; exported CSV tables; report files

**Table 3:** Methodological rationale for selected workflow decisions.

All-gaze/fixation file-pair validation	Prevents analysis from proceeding when participant-level exports are missing, duplicated, or unmatched.	File-pair status table; problem-file summary	Cannot determine whether a structurally complete recording is scientifically usable.
Sampling-rate diagnostics	Checks whether exported timestamps are consistent with the expected recording stream.	Participant-by-media sampling summaries	Does not independently verify vendor hardware specifications.
Tracking-quality review flags	Identifies recordings with low gaze validity, missingness, or out-of-range coordinates for user inspection.	Tracking-quality tables; review-required indicators	Thresholds are decision aids and require study-specific justification.
Pupil artefact handling and interpolation branches	Separates raw missingness, artefact removal, interpolation, smoothing, and baseline correction.	Processing flags; retained-sample counts; pupil-window summaries	Interpolation suitability depends on blink structure, task timing, and analysis windows.
AOI denominator audits	Makes spatial classification and proportion denominators inspectable before AOI modelling.	AOI summaries; denominator checks; fixation/AOI transition outputs	Does not determine whether the substantive AOI design is theoretically valid.
Exclusion recommendations	Converts diagnostic outputs into transparent review candidates without enforcing automatic deletion.	Participant- and trial-level recommendation tables	Final exclusion decisions remain design- and hypothesis-dependent.
Reporting checklists	Documents preprocessing, quality-control, and analysis-readiness decisions for manuscript reporting.	Checklist and analysis-decision audit objects	Completeness of reporting still depends on user interpretation and study context.

**Table 4:** Evidence level for major gp3tools workflow components.

Workflow Component	Evidence Reported in This Manuscript	Claim Supported	Claim not Supported
File discovery and all-gaze/fixation pairing	Automated tests, synthetic export folder, private real-export compatibility check	The workflow can detect and pair expected Gazepoint-style files under tested conditions.	Robustness across all Gazepoint software versions, naming conventions, or non-standard export structures.
Sampling and tracking-quality diagnostics	Automated tests and synthetic workflow outputs	Diagnostic tables and review flags are generated and preserved.	Independent verification of hardware sampling accuracy, gaze accuracy, or empirical tracking validity.
Pupil preprocessing branches	Automated tests and synthetic workflow outputs	Raw, cleaned, interpolated, baseline-corrected, and window-level outputs can be produced and compared.	Ground-truth recovery of true pupil size or universal optimality of preprocessing choices.
AOI, fixation, and transition workflows	Synthetic workflow outputs and structured object checks	AOI summaries, fixation summaries, and transition structures can be generated from available labels/events.	Validation of AOI theory, fixation-detection accuracy, or empirical scanpath effects.
Model-preparation and reporting outputs	Automated tests, synthetic outputs, report generation, and optional-dependency checks	Model-ready and report-ready objects can be produced for downstream analysis.	Validity of any specific statistical model, hypothesis test, or empirical conclusion.
Interoperability outputs	Adapter-object generation and optional external-package checks	Gazepoint-derived objects can be structured for related R workflows under tested conditions.	Long-term compatibility with all future external package versions.

**Table 5:** Reproducible demonstration workflow used to execute the synthetic Gazepoint-style example.

Step	R Command	Purpose
1	library(gp3tools)	Loads the gp3tools package.
2	demo_dir <- system.file (“extdata”, “gazepoint_realistic_demo_exports”, package = “gp3tools”)	Locates the synthetic Gazepoint-style demonstration export folder distributed with the package.
3	workflow <- run_gazepoint_workflow (input_dir = demo_dir, output_dir = “gp3tools_demo_output”, prefix = “gp3tools_demo”, save_plots = TRUE, create_report = TRUE)	Runs the end-to-end Gazepoint workflow, saves diagnostic plots, exports output tables, and creates a reproducible report.
4	summarise_gazepoint_workflow (workflow)	Summarises the generated workflow object, including imported rows, quality-control outputs, AOI outputs, plot files, and report status.

**Table 6:** Synthetic export folder and workflow summary.

Output	Count	Interpretation
Synthetic all-gaze files	48	Participant-level sample exports
Synthetic fixation files	48	Participant-level fixation exports
Summary exports	1	Gazepoint-style summary output
Complete file pairs	48	Expected all-gaze/fixation pairs detected as complete
Problem file pairs	0	No missing or duplicated pairs in the synthetic folder
All-gaze rows	115,200	Sample-level gaze and pupil observations
Fixation rows	5691	Fixation-level observations
Output table files	4	CSV outputs generated by the workflow
Output plot files	2	Diagnostic plots generated by the workflow
Report created	Yes	HTML diagnostic report created

**Table 7:** Tracking-quality summary by synthetic condition.

Condition	Recordings	Review-Required	Review-Required %	Mean Gaze-Valid %	Minimum Gaze-Valid %	Maximum Gaze-Valid %
Control	48	12	25.00	95.21	84.83	98.33
Distractor-heavy	48	21	43.75	92.71	85.00	96.00
Early-target	48	16	33.33	94.81	84.83	98.00
Late-target	48	11	22.92	94.65	84.33	97.83

**Table 8:** Pupil workflow outputs in the synthetic demonstration.

Object	Status	Rows	Interpretation
Raw pupil windows	Completed	768	Window-level pupil summaries from the raw branch
Linear-interpolated pupil windows	Completed	768	Window-level summaries after linear interpolation
PCHIP-interpolated pupil windows	Completed	768	Window-level summaries after PCHIP interpolation
Pupil-window model data	Completed	767	Confirmatory model-ready pupil-window table
Pupil-window LMM	Completed	—	Representative linear mixed-model object
Pupil-window sensitivity	Completed	—	Sensitivity comparison across pupil branches
Pupil GAMM	Completed	—	Time-course pupil modelling object
PFE-GAMM sensitivity	Completed	—	Pupil foreshadowing-effect sensitivity object

**Table 9:** Fixation and transition workflow outputs.

Output	Status	Rows	Interpretation
Fixation data	Completed	5691	Fixation-level observations
Fixation-trial summaries	Completed	192	Trial-level fixation summaries
AOI entries	Completed	96,572	AOI state-entry sequence rows
AOI transitions	Completed	192	Trial-level transition summaries
Transition events	Completed	96,380	Transition-level event data
Transition-count data	Completed	10,602	Count data for transition sensitivity modelling
AOI transition matrix	Completed	—	Count and probability transition matrices
Time-varying transition matrix	Completed	—	Windowed transition matrices
Markov-style object	Completed	—	Markov-compatible transition representation
HMM-ready data	Completed	—	Sequence and emission-ready data
Semi-Markov-ready data	Completed	—	State-sequence and dwell-time data

**Table 10:** Reporting and interoperability outputs.

Output	Status	Rows	Interpretation
Real-data readiness gate	Completed	—	Structured readiness assessment
Exclusion recommendations	Completed	—	Participant- and trial-level review recommendations
Reporting checklist	Completed	—	Manuscript/reporting readiness checklist
Analysis-decision audit	Completed	—	Summary of workflow decisions and cautions
eyetools adapter data	Completed	115,200	Data prepared for eyetools-style workflows
gazer adapter data	Completed	115,200	Data prepared for gazer-style workflows
PupillometryR-style adapter data	Completed	115,200	Data prepared for pupil-analysis workflows
eyetrackingR-style adapter data	Completed	115,200	Data prepared for eyetrackingR-style workflows
gazeR cross-check	Completed	—	Optional external-package cross-check
eyetools fixation detection	Completed	—	Optional fixation-detection check

**Table 11:** Summary of synthetic demonstration outputs.

Output	Count	Interpretation
All-gaze rows	115,200	sample-level gaze and pupil observations
Fixation rows	5691	fixation-level observations
Sampling-quality rows	192	participant-by-media sampling diagnostics
AOI summary rows	1344	area-of-interest summaries across trials/windows
Review-required rows	60	rows flagged for review rather than automatic deletion
Complete file pairs	48	all expected all-gaze/fixation pairs found

**Table 12:** Software-quality evidence reported for gp3tools.

Evidence	Role
Automated tests	Check core functions, edge cases, optional-dependency paths, and workflow objects
Package checks	Confirm package installation, documentation, examples, vignettes, and R CMD check compliance
Cross-platform continuous integration	Evaluate package behaviour across major operating systems and R release/development settings
Release archiving	Provide a persistent versioned software citation through Zenodo
Documentation	Provide README guidance, function help, vignettes, example data, and case-study workflows
Synthetic demonstration	Exercise the export-to-report workflow without exposing private participant recordings

## Data Availability

The manuscript uses synthetic Gazepoint-style demonstration data distributed with the public gp3tools repository: https://github.com/stefanosbalaskas/gp3tools. No private participant-level eye-tracking recordings are redistributed with this article. Researchers applying the package to empirical Gazepoint recordings should report study-specific ethics approval, consent procedures, calibration protocol, device settings, exclusion rules, preprocessing decisions, and model specifications.

## Supplementary Materials

The gp3tools source code, documentation, tests, vignettes, example datasets, and synthetic Gazepoint-style demonstration materials are publicly available at the GitHub repository: https://github.com/stefanosbalaskas/gp3tools. Version 1.0.1 is archived on Zenodo with DOI 10.5281/zenodo.20731340. The package is distributed under the MIT licence.

## Abbreviations

The following abbreviations are used in this manuscript:

AOI	Area of interest
CSV	Comma-separated values
GAMM	Generalised additive mixed model
GLMM	Generalised linear mixed model
GP3	Gazepoint GP3 eye tracker
HMM	Hidden Markov model
LMM	Linear mixed model
PCHIP	Piecewise cubic Hermite interpolating polynomial
R	R statistical computing environment
